# Assessing elderly’s functional balance and mobility via analyzing data from waist-mounted tri-axial wearable accelerometers in timed up and go tests

**DOI:** 10.1186/s12911-021-01463-4

**Published:** 2021-03-25

**Authors:** Lisha Yu, Yang Zhao, Hailiang Wang, Tien-Lung Sun, Terrence E. Murphy, Kwok-Leung Tsui

**Affiliations:** 1 School of Public Health (Shenzhen), Sun Yat-Sen University, Guangdong, People’s Republic of China; 2 School of Data Science, City University of Hong Kong, Kowloon, Hong Kong; 3 School of Design, The Hong Kong Polytechnic University, Kowloon, Hong Kong; 4 Department of Industrial Engineering and Management, Yuan Ze University, Taoyuan, Taiwan; 5 Department of Internal Medicine, Yale University School of Medicine, New Haven, USA; 6 Department of Industrial and Systems Engineering, Virginia Tech, Blacksburg, USA

**Keywords:** Balance and mobility, Fall, Elderly care, Sensor, Data mining, Timed up and go

## Abstract

**Background:**

Poor balance has been cited as one of the key causal factors of falls. Timely detection of balance impairment can help identify the elderly prone to falls and also trigger early interventions to prevent them. The goal of this study was to develop a surrogate approach for assessing elderly’s functional balance based on Short Form Berg Balance Scale (SFBBS) score.

**Methods:**

Data were collected from a waist-mounted tri-axial accelerometer while participants performed a timed up and go test. Clinically relevant variables were extracted from the segmented accelerometer signals for fitting SFBBS predictive models. Regularized regression together with random-shuffle-split cross-validation was used to facilitate the development of the predictive models for automatic balance estimation.

**Results:**

Eighty-five community-dwelling older adults (72.12 ± 6.99 year) participated in our study. Our results demonstrated that combined clinical and sensor-based variables, together with regularized regression and cross-validation, achieved moderate-high predictive accuracy of SFBBS scores (mean MAE = 2.01 and mean RMSE = 2.55). Step length, gender, gait speed and linear acceleration variables describe the motor coordination were identified as significantly contributed variables of balance estimation. The predictive model also showed moderate-high discriminations in classifying the risk levels in the performance of three balance assessment motions in terms of AUC values of 0.72, 0.79 and 0.76 respectively.

**Conclusions:**

The study presented a feasible option for quantitatively accurate, objectively measured, and unobtrusively collected functional balance assessment at the point-of-care or home environment. It also provided clinicians and elderly with stable and sensitive biomarkers for long-term monitoring of functional balance.

## Background

Falls among elderly have been cited as a serious health issue that results in physical and psychological trauma and thus increases pressure on healthcare systems. Approximately one-third of people over 65 years old fall each year, with the fall rate increasing with age [1]. For example, 30–60% of the community-dwelling elderly in the U.S fall each year, and more than 50% of them experience multiple falls [2]. In Hong Kong, the prevalence of falls among the community-dwelling elderly is around 18–19%, but it is believed that many cases are not reported [3]. Approximate 25–35% of elderly residents in Taiwan experience fall-related injury more than one time per year [4]. Falls can result in lasting and critical consequences, including injury leading to hospitalization, reduced activity and mobility level, fear of falling and even death. In light of these adverse consequences, efforts to prevent the occurrence of falls and their subsequent impacts have been undertaken. An effective fall prevention program first identifies those elderly at highest risk of falling, and subsequently determines the most appropriate interventions, with a goal of first preventing a fall and secondly of reducing its severity. There is evidence in the fall prevention literature which suggests that more than 50% of potential falls relating to elderly could be avoided with systematic implementation of fall prevention interventions [5].

There are various driven factors of falls. Poor balance has been validated in the literature as one of the key causal factors of falls among elderly, and the continuous monitoring of gait and balance is a plausible approach to reduce and prevent falls through early warnings and appropriate interventions [6]. However, the continuous monitoring of gait and balance requires extensive healthcare and clinical resources. Limited professional resources (e.g., physical therapists) versus growing aging population worldwide are insufficient to detect balance deteriorations in a timely fashion and thus could result in many falls that could have been avoided through continuous monitoring and early interventions. In order to fill in such a gap between resources and care needs, it is urgently needed an approach for timely assessing balance among the community-dwelling elderly without healthcare professionals’ involvement. We therefore propose to supplement professional assessment by developing a surrogate assessment approach of balance that uses sensor technology and statistical data mining.

**Fig. 1:**
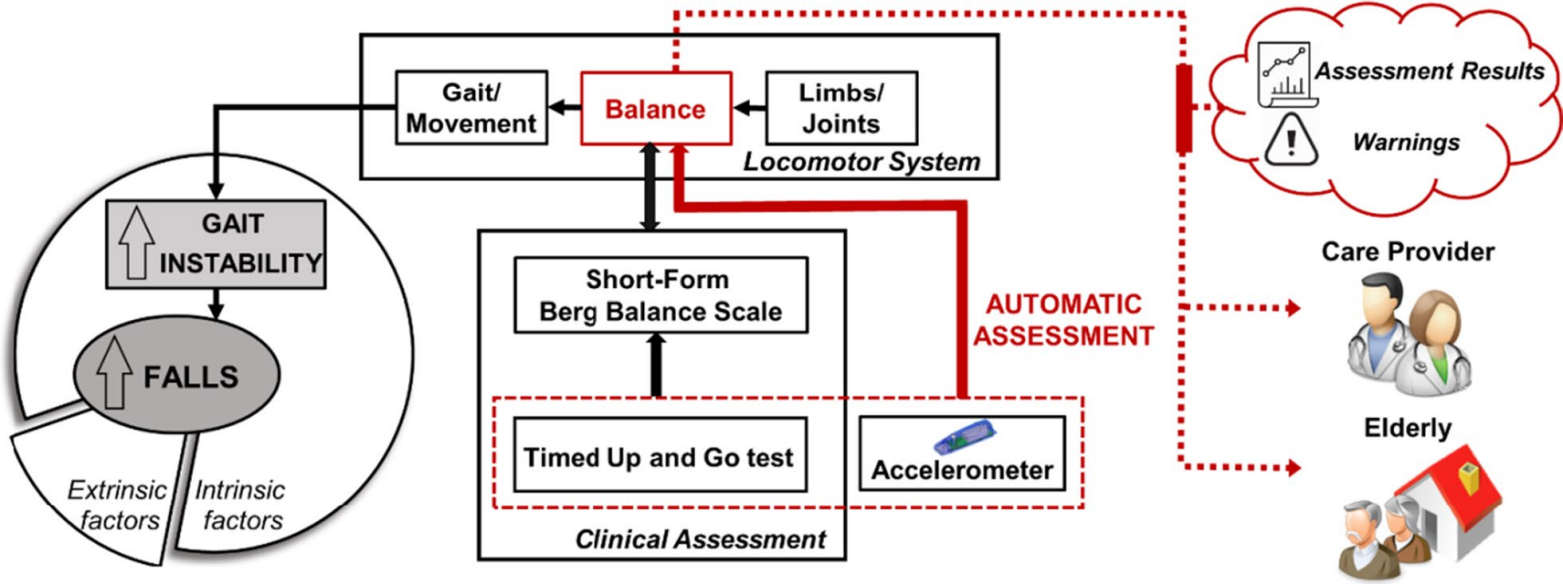
The novel clinical perspectives of our study

In this paper, we investigated the effectiveness of the 3-m Timed Up and Go (3MTUG) walking test via a waist-mounted tri-axial accelerometer in estimating the Short Form Berg Balance Scale (SFBBS) for assessing a community-dwelling elderly’s functional balance. We believe this approach functions well in three domains; it is quantitatively accurate, objectively measured, and unobtrusively collected. Figure 1 illustrates the clinical perspective of our study. This combination of the research setting, study population, and clinical context represents a novel application of wearable-sensor based fall risk assessment. The automatic estimation of functional balance using computerized system and wearable-sensor can bring more sensitive, specific and responsive balance testing to clinical practice.

This paper advances the application of wearable sensor in the field of elderly fall risk assessment. In our literature review in the next section, we note that the existing literatures on sensor-based fall risk assessment presents over-optimistic results mainly due to the curse of dimensionality, improper model selection and lack of validation [7]. While accounting for our problem formulation and limitations in existing literature, in this work, we focus on developing a clinically relevant, effective and robust approach to assessing the functional balance of the elderly in terms of SFBBS. The major scientific contributions of this paper are threefold. Firstly, there are infinitely multiple ways to extract features from accelerometer signals and no consensus achieved in existing literature regarding the optimal accelerometer-based variables for modeling. In this work, we adopt significant variables that have previously been associated with fall-risk in elderly with an emphasis on the selection of clinically relevant variables. We strive to derive meaningful variables by incorporating prior information.

Furthermore, we propose a new approach to extract these significant variables by separate considering postural transitions and walking phases during the 3MTUG walking test. Our proposed adoption of these clinically accelerometer-based variables will allow the findings of this study to both consolidate previous research findings and build upon these findings with original and clinically focused contribution. Secondly, using proposed accelerometer-based variables as input and considering SFBBS score as the reference measure of an elderly’s functional balance, elastic net regression together with random-shuffle-split cross-validation strategy is developed to avoid model overfitting and enhance generalization. Thirdly, we provide an in-depth identification of frequent selected and relatively important variables that relate to the functional balance of an elderly using our proposed model. This identification is helpful to determine the underlying motor mechanisms contributing to balance disorders. We also investigate an elderly’s balance risk level in the performance of three selected clinical assessment tasks, which may facilitate the diagnostics and treatment procedures of balance disorder in clinical practice.

The remainder of this paper is organized as follows. Section 2 illustrates an extensive literature review about falls and sensor technologies. Section 3 shows the experimental design for data collection. Section 4 describes the methodology for data analysis. Section 5 presents our results and discussion of key findings. Finally, Sect. 6 presents conclusions and implications.

## Literature review

Falls can be caused by a complex interaction among intrinsic and extrinsic risk factors. Intrinsic, or patient-related, risk factors related to natural aging changes that affect elderly's physical ability, vision, muscle strength and changes in their cognition. For example, it includes increasing age, gait and mobility disorder, balance impairment, muscle weakness, history of falls, medical conditions, and cognitive impairment [8]. Extrinsic factors include factors that are external to an elderly's functional ability, physical health, and cognition. For example, these factors include inadequate lighting, wet floor surfaces, loose carpets, slippery handrails, and inappropriate footwear and clothing [9]. Poor balance has been validated in the literature as one of the key causal factors of falls among elderly [6]. In this paper, we limit our scope primarily to the balance, which is important for a stable body position and thus prevention of falls.

Balance refers to an ability to control the center of gravity over the base of support [10]. It is also described as an individual’s ability to respond to a sudden perturbation caused by extrinsic or intrinsic factors. The control of balance is complex, with strong integration and coordination of multiple body elements including visual, vestibular and somatosensory system. Deterioration of functional capacities associated with age leads to balance impairment. Physiological deficits secondary to aging, such as impaired balance, decreased muscle function and limited joint mobility, may perturb the locomotor system of elderly to bring about gait instability [11]. Gait instability is well identified as a significant risk factor leading to falls and has been recognized as a measure for identifying potential fallers [12]. The assessment of balance is, therefore, one of the primary measures for the prevention of falls, together with subsequent multifactorial assessment and intervention programs.

To assess balance, clinicians rely mostly on comprehensive medical and functional mobility assessment tools in the form of physical test, gait analysis and physical activity measurement [13]. A number of validated assessment tests in a clinical setting have been developed, such as SFBBS [14] and Timed Up and Go (TUG) walking test [15]. SFBBS is a tool used to assess static and dynamic balance abilities. It is a simplified version of the well-known Berg Balance Scale (BBS) [16], which was adopted as the ‘gold standard’ of balance performance. It is used to classify fallers and non-fallers with a sensitivity and specificity of 82.5% and 93% respectively [17]. The TUG test has been extensively researched and is being widely used in clinics to assess balance and mobility for over 20 years. It quantifies several different mobility elements and has been utilized in the fall risk assessment frequently [18]. Although these kinds of assessments allow for comprehensive quantitative comparisons of performance on diverse tasks, the majority of these assessments are restricted to use in a clinical environment, as their correct execution often requires supervision. Thereby, these tests are usually inappropriate for long-term monitoring of large patient cohorts under real-life conditions.

Recent advances have seen technology being increasingly used to assess functional balance. Researchers have investigated the potential use of sensors for objectively evaluating functional balance, as it could be less expensively, less time-consuming, more user-friendly and more feasible to use [19]. Despite this, the use of sensor-based assessment has far been limited. Firstly, most of current studies focused on the inpatients in a hospital or geriatric clinic while less attention was given to the community-dwelling elderly. Secondly, instead of classifying elderly using their fall history, few studies focused on regression problem and tried to replicate the score of validated clinical assessment tool. Under this category, previous studies used accelerometer signals acquired from Directed Routine (DR) to estimate the clinical assessment scores, such as BBS or Physiological Profile Assessment (PPA) [20–22]. However, in these studies, a very large pool of variables was constructed and it was hard to explain selected variables’ clinical relevance. In addition, the reported models were over-fitted and were not validated properly. Their variable selections were done either using the whole data set outside the cross-validation loop or using repeated cross-validation with the same data. The repeated cross-validation suffers the issue of inter-fold dependence of data and the negative bias introduced by only training on $K - {1}$ folds, especially if the model is unstable [7].

Motivated by these research gaps, in this study, we focus on exploring the use of clinically relevant accelerometer-based variables in effective estimation of the SFBBS score for assessing a community-dwelling elderly’s functional balance quantitatively, objectively and unobtrusively during the performance of the 3MTUG walking test.

## Experimental design

### Participant recruitment

Community-dwelling people aged > 65 years or older were recruited from the central area of Taiwan between April 2014 and May 2015. The inclusion criteria of the participants were as follows: no history of lesions of the central nervous system, no injuries in the musculoskeletal system, able to walk independently with or without any assistive devices within the last three months. A total of 85 participants (18 men and 67 women) aged 65–109 years (72.12 ± 6.99 year) were recruited. The study was approved by the Institutional Review Board of Tsaotun Psychiatric Center, Ministry of Health and Welfare, and written informed consent was obtained from participants prior to their participation.

### SFBBS

BBS is a widely used tool to assess the balance ability of geriatric people and geriatric patients [16]. Although this scale is highly reliable and valid, it takes time to complete, consists of five-level items with scoring criteria varying from item to item, and exists item redundancy. To simplify and improve its utility, the SFBBS was developed to include 7 main items from the original BBS, see Table 1 for details. Compared to BBS, only half the time (about 10 min) is required to complete all items. Studies had provided strong evidence suggesting that the SFBBS featured psychometric properties similar to those of the original BBS, and there was a good test–retest reliability (ICC = 0.95) of the SFBSS in elderly [14, 23]. The SFBBS has 3 categories for each 7 item, scored 0, 2, or 4 for a maximum total score of 28, with higher scores indicating better balance. The SFBBS is considered as the reference measure of an elderly’s balance in this paper.

**Table 1:** Item descriptions of the short form berg balance scale

Item	Description
1	Sitting to standing
2	Standing unsupported with eye closed
3	Reaching forward with an outstretched arm while standing
4	Pick up the object from the floor from a standing position
5	Turning to look behind over left and right shoulders while standing
6	Standing unsupported one foot in front
7	Standing on one leg

### 3-m timed up and go test

**Fig. 2:**
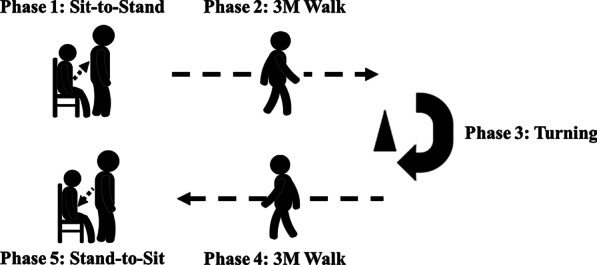
The illustration of the 3MTUG test

TUG test is one of the most widely used and accepted clinical tests for assessing functional mobility [15]. This is a simple test and easy to administer anywhere and anytime. It is made up of a set of basic mobility skills key to independent living and has been suggested as a useful screening tool for identifying elderly with balance or gait deficits. Figure 2 demonstrates the typical phases of the 3MTUG walking test. It is performed by standing from a seated position (phase 1), walking to a marker on the floor three meters away (phase 2), turning around 180° (phase 3), returning to the chair (phase 4), and returning to a seated position (phase 5).

### Procedure

The participants first self-reported their demographical information (e.g., age and sex) and were evaluated for their functional balance by a registered physiotherapist using the SFBBS. Before they performed the 3MTUG test, our research assistants helped to attach a wireless tri-axial accelerometer (Freescale RD3152MMA7260Q, Freescale Semiconductor-NXP, Austin, TX, USA) on the participants’ lower back in the area between the L3 to L5 vertebrae. This location was chosen for the sake of simplicity and it approximates the center-of-mass (CoM) [19]. Accelerometers were aligned with the vertical (V) (downward: positive), medial–lateral (ML) (right: positive), and anterior–posterior (AP) axes (anterior: positive). Accelerometer data were collected with a nominal sampling rate of 45 Hz. The data was steamed over a Bluetooth radio to a nearby laptop where a self-developed tailor-made application was used for data logging.

For the 3MTUG test, each participant began seated in an armchair with their back against the chair and was instructed to complete the test at a safe and comfortable pace. A research physical therapist also measured the time to completion of the TUG using a stopwatch. The time was measured from the moment the participants start to lean to the moment the participants sit back on the chair. No physical assistance was provided, but the participants could use their own walking aid if needed.

## Analytical methodology

### TUG task segmentation

We considered the overall TUG performance into two major phases: postural transition and walking. The postural transition refers to the tasks of sit-to-stand (SiSt) and stand-to-sit (StSi) transitions. Indeed, rising to a stand and controlling the descent to sitting are regarded as fundamental activities of daily activities [24], and are prerequisites for walking and standing [25]. These activities are not simply movements but have been widely recognized as the most mechanically and muscle demanding body movement tasks [26]. In particular, the elderly experience notable difficulties when performing these transitions due to their generally reduced balance and mobility.

**Fig. 3:**
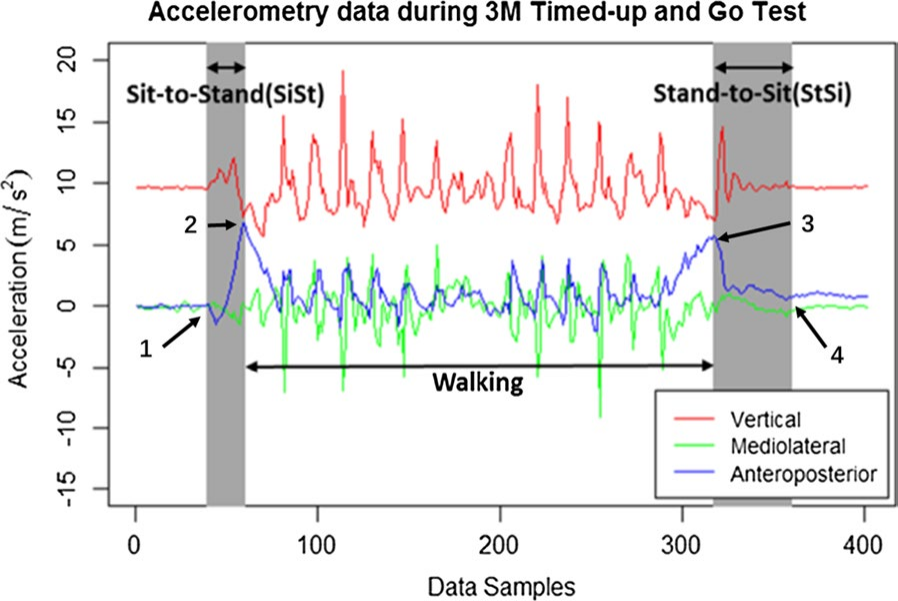
An illustrated example of segmented TUG tasks using accelerometer data

To achieve the segmentation, the collected accelerometer signal was first calibrated using Moe-Nilssen’s calibration algorithm, which transforms the data to the horizontal-vertical coordinate system [27]. Figure 3 shows an illustrated example of the following segmented task. In a typical accelerometer signal during the TUG task, key features of each separate phases can be seen. A clear “M”-shaped signal is identified in the AP axis except for the walking portion in between the SiSt and StSi components [28]. The first hill of the “M” shape reflects the SiSt component, while the second hill reflects the StSi component. These postural transitions are shaded in grey. The period of SiSt (interval 1–2) was defined as the time interval between point 1, the time of the signal started to rise from steady state, and point 2, the time of the first M-like maximum peak. The period of StSi (interval 3–4) was defined as the time interval between point 3, the time of second M-like maximum peak, and point 4, the time of the signal reached steady state. The walking phase is regarded as the interval between maximum peaks, i.e., points 2 and 3. These definition was elected because they were the easiest to derive reliably and consistent among subjects. A detail discussion can be found in [29].

Our identified two major TUG phases are similar to those adopted in previous studies of instrumented TUG performance where five phases were considered [28, 30]. The segmentation aims to capture the different characteristics of different body movements. The primary consideration for the postural transition is related to muscle power, lower limb strengths, and joint range while that for the walking is related to stability, symmetry, and regularity. The clinical relevance of analyzing the performance of each phase of the TUG is that it may be used to highlight specific areas of difficulty in task performance on an individual basis, and thus to inform subsequent intervention [31].

### Features extraction and construction

Current literature has identified a wide range of accelerometer-based features, but with no consensus regarding the optimal variables to examine from the data obtained. The chosen features, or the extraction manner, often differ among studies. In this work, we aimed to extract a combination of features which have previously been associated with fall-risk in the elderly, with an emphasis on the selection of clinically relevant variables. According to the recent review study [32], the frequency of features reported from different combinations of functional task, sensor placement, and feature category vary greatly. The number of studies on walking task is approximately eight times than that on postural transition task. Thereby, to select significant features in our study, different inclusion criteria were applied to each segmented TUG phases based on our data availability. Features were selected for the postural transition if they were reported significantly (*p* < 0.05) regardless of participants’ pathological condition and accelerometer sensor placement. Features were selected for the walking if: [feature was reported significantly in at least two studies (*p* < 0.05)] AND [feature was computed for walking task] AND [accelerometer was used, and it was worn on the lower back/trunk] OR feature was independent of sensor placement and type (e.g., number of steps). Our selection of features was mainly based on the papers [19, 28, 32–36]. These features were further categorized similarly to [19]: linear acceleration, spatial, temporal, frequency and other. Subsequently, redundant features were removed. The selected linear acceleration features were considered for all three axes respectively (V, ML, AP). Table 2 summaries the selected features extracted from different segmented TUG tasks in our study.

1. *TUG*: The temporal feature includes the duration of the overall TUG performance {1} [28].
2. *SiSt*: The first part of the linear acceleration features is the amplitude descriptive statistics: maximum {2–4}, minimum {5–7}, range {8–10}, root-mean-square (RMS) {11–13} for each three axes respectively. The others are jerk-related features. The jerk indicates the rate of change in acceleration. All slope are calculated after making a linear fit in the relevant intervals. In the case of SiSt, the jerk is divided into two parts where the first part represents the leaning forward of the trunk when preparing to rise from the chair and the second part represents the beginning of the active rise (while stilling is leaning forward) [29]. The maximum {14–16} and mean {17–19} of jerk are calculated from those two parts. Δjerk {20–22} is defined as the difference between two part jerks. The temporal feature includes the SiSt duration {23}.
3. *Walking*: The linear acceleration features are the RMS amplitudes {24–26}. To provide an indication of the repeatability of acceleration pattern from step to step, signals were divided into individual steps by identifying peak detection in the vertical axis. The average step length {27} is calculated by dividing the total number of steps with total length (6 m). Cadence {28} can be calculated as 60 times the number of steps taken divided by the walking phase duration. Gait speed {29} is calculated by dividing the total length by the walking duration. The coefficient of variation (CV) for step-time {32} and the CV of stride time {33} are calculated to provide a measure of gait variability as the ratio of the standard deviation to the mean of step time {30} and stride time {31}. Three common frequency features are also included for each of the three axes. Dominant Fourier transform frequency {34–36} is the dominant frequency on a Fast Fourier Transformation (FFT) frequency plot. The first quartile of FFT is a percentage of acceleration frequencies within the first quartile of an FFT frequency plot {37–39}. A lower value has been linked to instability [37]. The ratio of even to odd harmonics (REOH) {40–42} reflects the proportion of the acceleration signal that is in phase with the participant's stride frequency.
4. *StSi*: Same interpretation apply to features {43–54} as those {2–13} from SiSt section. For jerk related features {55–63}, in the case of StSi, the first part of the jerk represents the lowering of the center of mass backward when leaning the trunk backward to sit on the chair and the second part represents the free fall. The temporal feature is the StSi duration {64}. To measure the temporal variation between SiSt and StSi postural transitions, the standard deviation of durations of these two phases is also considered {65}.
5. *Other*: Two available demographic features age {66} and gender {67} are considered.

**Table 2:** Summary of the 67 features by task and feature category

Accelerometer-based features					
TUG					
*Temporal*					
	1	Duration			
Sit-to-stand (SiSt)					
*Linear acceleration features (in the order of V, ML, AP)*					
	2–4	Maximum		5–7	Minimum
	8–10	Range		11–13	RMS
	14–16	Maximum jerk		17–19	Mean jerk
	20–22	Δjerk			
*Temporal*					
	23	Duration			
Walking					
*Linear acceleration features (in the order of V, ML, AP)*					
	24–26	RMS			
*Spatial features*					
	27	Step Length			
*Temporal*					
	28	Cadence		29	Gait speed
	30	Step time		31	Stride time
	32	CV of step time		33	CV of stride time
*Frequency features (in the order of V, ML, AP)*					
	34–36	Dominant FFT peak		37–39	1st FFT
	40–42	REOH			
Stand-to-sit (StSi)					
*Linear acceleration features (in the order of V, ML, AP)*					
	43–45	Maximum		46–48	Minimum
	49–51	Range		52–54	RMS
	55–57	Maximum jerk		58–60	Mean jerk
	61–63	Δjerk			
*Temporal*					
	64	Duration		65	SD (SiSt, StSi)
Demographic features					
*Other*					
	66	Age		67	Gender

### Prediction models

Predictive modeling is a data-mining tool used to correlate a response variable with a set of predictor variables. Depending on whether the response variable is categorical or not, the prediction task can be formulated as a classification or regression problem. Regularized regression models and decision tree were selected to use for their effectiveness of prediction in the literature. Besides, the parameters of the fitted model can be interpreted easily to provide significance of each feature.

- Regularized regression

The elastic net regression is a powerful and versatile model [38]. It is a regularization method for fitting a generalized linear model (GLM). The standard ordinary least squares (OLS) regression performs poorly on high dimensional data sets, where there is a large multivariate dataset containing a relatively large number of variables to the number of samples. Regularized regression techniques have been created the last few ten years to reduce the flaws of OLS regression with regard to prediction accuracy. It is known that the ridge penalty [39] shrinks the coefficients of correlated predictors towards each other while the lasso [40] tends to pick one of them and discard the others. The elastic net penalty mixes these two.

The objective function of the elastic net regression takes the form of ‘loss + penalty’:

$${\text{arg}}\mathop {\min }\limits_{\beta } {\varvec{y}} - {\mathbf{X}}{\varvec{\beta}}_{2}^{2}$$

1 $${\text{s.t.}}\quad \left( {1 - \alpha } \right)/2\beta_{2}^{2} + \alpha \beta_{1}^{{}} \le t,$$

where $\alpha$ is the elastic net penalty which controls the balance between the ridge and lasso regression, $\beta_{2}^{2} = \sum\nolimits_{j = 1}^{p} {\beta_{j}^{2} }$ is the L2-norm of the $\beta$, $\beta_{1} = \sum\nolimits_{j = 1}^{p} {\left| {\beta_{j} } \right|}$ is the L1-norm of $\beta$ and $t$ is a tuning parameter. The elastic net regression clarifies to simple ridge regression when $\alpha = 0$ and to the lasso regression when $\alpha = 1$. In this paper, we applied three regularized regressions, including ridge, lasso and elastic net regression for predicting the SFBBS score.

- Decision tree

Decision Tree is a popular method that is simple and easy to implement [41]. It requires no domain knowledge or parameter setting. It constructs hierarchical decision trees by splitting data among classes of the criterion at a given node accordingly to an “if–then” rule applied to a set of predictors, into two child nodes repeatedly, from a root node that contains the whole sample. It selects the input variable that has the strongest association with the dependent variable according to a specific criterion. For a regression problem, the splitting criteria is to maximize

2 $$SS_{A} - \left( {SS_{{A_{L} }} + SS_{{A_{R} }} } \right),$$

where $SS_{A} = \sum \left( {y_{i} - \overline{y}} \right)^{2}$ is the sum of squares for the node $A$, and $SS_{{A_{R} }}$, $SS_{{A_{L} }}$ are the sums of squares for the right and left sub-nodes, respectively. This is equivalent to choosing the split to maximize the between-groups sum-of-squares in a simple analysis of variance. The built decision tree produces a model that represents interpretable rules or logic statements.

### SFBBS prediction

Our SFBBS score estimation was a typical prediction problem. We defined the input of the predictive model was the features (Table 2) extracted from the accelerometer signal during the TUG performance. The output of the model was the numerical value of the SFBBS score.

- Evaluation metrics

Two metrics were employed to measure the prediction accuracy: mean absolute error (MAE) and root mean square error (RMSE). MAE measures the average magnitude of the errors without considering their directions. On the other hand, RMSE is a quadratic scoring rule that also measures the average magnitude of the error, and it is sensitive to large errors. For a set of predicted values ($\hat{y}_{1} , \hat{y}_{1} , \ldots , \hat{y}_{n}$) and the corresponding observed values ($y_{1} , y_{2} , \ldots , y_{n}$),

3 $${\text{MAE}} = \frac{1}{n}\mathop \sum \limits_{j = 1}^{n} |y_{j} - \hat{y}_{j} |$$

4 $${\text{RMSE}} = \sqrt {\frac{1}{n}\sum\nolimits_{j = 1}^{n} {\left( {y_{j} - \hat{y}_{j} } \right)^{2} } } ,$$

where smaller MAE and RMSE values indicate better prediction performance.

### SFBBS subtask prediction

**Fig. 4:**
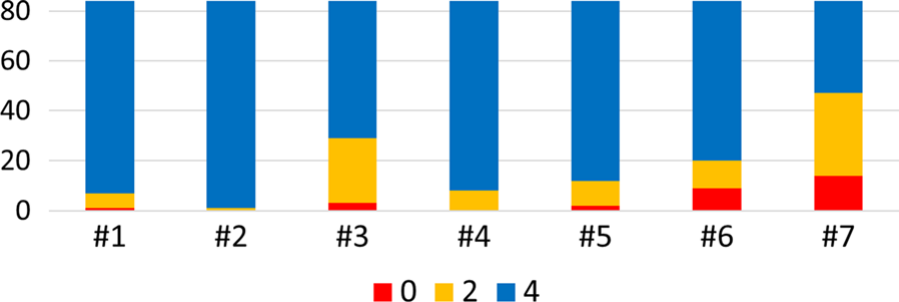
The score distribution of the participants’ SFBBS subtasks

The control of body equilibrium is complex and cannot be evaluated fully with any global measurement of “balance” [42]. Based on the availability of the scores on each SFBBS subtasks, we plot the score distribution of the participants’ SFBBS each subtask as shown in Fig. 4. As per expectation, variations in the distribution of score are observed across different SFBBS subtasks. For example, every participant get score 4 in task #2 while one third get score 0 in task #7. It is interesting to explore what affecting the difference scoring in each task. We thus investigate the key features that are related to different assessment tasks. Three tasks with large variation in scoring are selected for subsequent analysis: task #3 (reaching forward with an outstretched arm while standing), task #6 (standing unsupported one foot in front), and task #7 (standing on one leg). The selected three tasks cover the evaluation of the restriction of joint range of motion, muscle strength and postural stability that can affect the maintenance of equilibrium positions. Our selection of these three tasks is consistent with previous literature [43], where these tasks have been identified as the most difficult items for elderly to perform successfully.

- Decomposition of SFBBS subtasks score

We first grouped the scores of selected three tasks into easily defined, clinically relevant subgroups. Score 0 and 2 were regarded as “High-risk”, and score 4 was regarded as “Low-risk”. Thereby, we turned the prediction problems into a typical binary classification problem. This categorization can lead to less biased and more informative response variable for identifying the high-risk elderly on performing certain movement tasks.

- Evaluation metrics

To measure the classification performance, we constructed a confusion matrix as illustrated in Table 3. The overall classification accuracy, precision, sensitivity, specificity, and F-score were used as performance metrics, as shown in Eqs. (6)–(10). Accuracy measures the proportion of observations classified correctly, both “High-risk” and “Low-risk”. The precision measures the proportion of true “High-risk” observations among classified “High-risk” observations. Sensitivity measures the proportion of “High-risk” that are correctly classified while specificity measures the proportion of “Low-risk” that are correctly classified. There is usually an inverse relationship between precision and sensitivity. F-score represents a harmonic mean between precision and sensitivity.

5 $${\text{Accuracy}} = \frac{{{\text{TP}} + {\text{TN}}}}{{{\text{TP}} + {\text{TN}} + {\text{FP}} + {\text{FN}}}}$$

6 $${\text{Precision}} = \frac{{{\text{TP}}}}{{{\text{TP}} + {\text{FP}}}}$$

7 $${\text{Sensitivity}} = \frac{{{\text{TP}}}}{{{\text{TP}} + {\text{FN}}}}$$

8 $${\text{Specificity}} = \frac{{{\text{TN}}}}{{{\text{FP}} + {\text{TN}}}}$$

9 $${\text{F - score}} = 2 \cdot \frac{{{\text{Precision}} \cdot {\text{Sensitivity}}}}{{{\text{Precision}} + {\text{Sensitivity}}}}$$

**Table 3:** Confusion matrix

	Reference (high-risk)	Reference (low-risk)
Predicted (high-risk)	True positive (TP)	False positive (FP)
Predicted (low-risk)	False negative (FN)	True negative (TN)

In addition, the Receiver Operating Characteristic (ROC) curve and the area under the ROC curve (AUC) were employed to measure the quality of the model’s performance. The ROC explicates the relation between true positive rate (i.e., sensitivity) and false positive rate (i.e., 100%-specificity) for various cut-offs of a continuous diagnostic test. The AUC reports an overall quantitative estimate of classification performance, with higher value indicates better performance ($0 \le AUC \le 1$).

### Model implementation and cross-validation

The aforementioned predictive models considered were directly applied to 85 elderly’s datasets for estimating their balance. To avoid over-fitting problems as well as minimize the bias, we used 100-iteration random-shuffle-split cross-validation (100-RSSCV). To do this, a single random-shuffle-split was configured to select a random subset of 80% of the data for training the model with the remaining 20% of the data as a random subset for model testing. This process was repeated for 100 iterations. Within each iteration, a new model was trained and validated from the training data using 10-fold cross-validation and tested on the testing data. The performance evaluation metrics were calculated from the 100 iterations. Our selected cross validation aimed to promote generalizability and reliability and avoided methodological problems associated with validation and training–testing protocols seen in the fall-risk assessment literature [7]. The chosen number of iterations was based on the coverage of the performance metrics and the selection of significant variables in our preliminary studies. All statistical analysis was implemented in R v3.4.1 (64 bit) using the “glmnet”, “rpart”, “caret” and “e1071” packages [44].

## Results and discussion

### SFBBS prediction

**Fig. 5:**
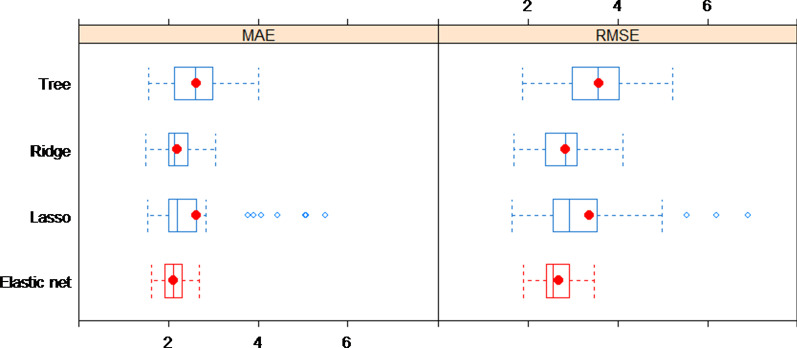
Box-whisker plots of the performance of four models by MAE and RMSE

In this section, we provide a comparison of the four methods’ performance on the task of SFBBS score estimation. Figure 5 shows the box-whisker plots of the performance achieved by the four models based on MAE and RMSE. In each plot, the central box represents the values from the lower to upper quartile (25–75 percentile). The middle line represents the median. The horizontal line extends from the minimum to the maximum value. Besides, we use a red dot to indicate the mean value. It is clear that the regularized regressions perform better than the decision tree. The elastic net regression (mean (std.) values of MAE and RMSE are 2.12(0.26) and 2.68(0.40) respectively) is superior with the smallest mean and variance in terms of both MAE and RMSE. The ridge and lasso regression show competitive results. We can see that while the ridge regression generally performs stable in the random-shuffle-split iterations, the lasso regression performs well in most of the iterations but perform poorly in some cases. Thereby, the performance of selected models in descending order is elastic net regression, ridge regression, lasso regression, and decision tree. This result is unsurprising since the elastic net regression considers both L1 and L2 norm penalty, where ridge or lasso regression is a special case of it. It means that if the ridge or lasso regression is indeed the best in one iteration, then the elastic net model selection routine will also identify that as the best model.

- Significant features

**Fig. 6:**
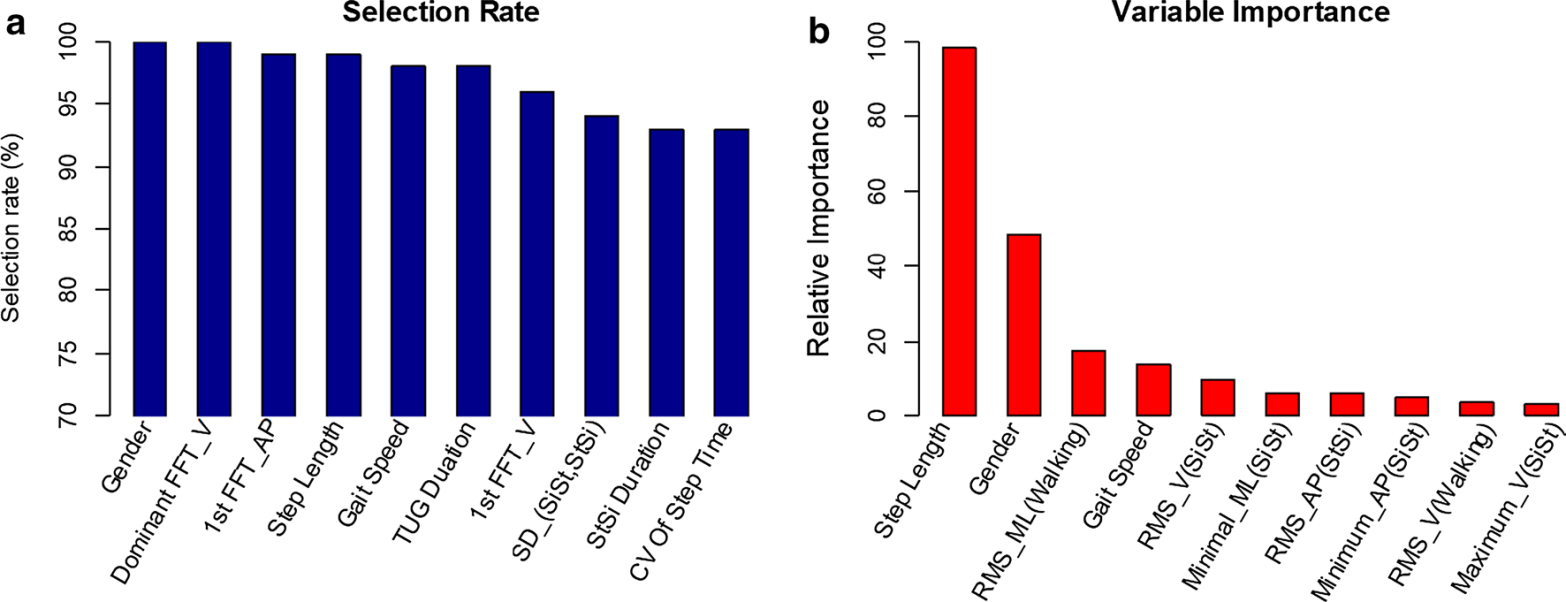
Histogram of selected features by elastic net regression using 100-RSSCV on SFBBS score estimation: **a** the top ten most selected features; **b** the top ten most significant features with respect to their accumulated regression coefficients

Additionally, we report the features which are significant to the balance using the results of elastic net regression. As described above, one set of features was selected for a new predictive model in each iteration of 100-RSSCV. Hence, the number of times each feature was selected during the 100-RSSCV can be calculated straightforward. Histograms of top ten selected features (out of 100 iterations) are shown in Fig. 6a. As can be seen from the figure, the most frequently occurring features in descending order of frequency are {67, 34, 39, 27, 29, 1, 37, 65, 64, 32}. Besides, we study the relative importance of features to get an insight of which features are most sensitive in terms of predicting the SFBBS. It is measured as the sum of regression coefficients of each feature during the 100-RSSCV. The top ten most relative features {27, 67, 25, 29, 11, 6, 54, 7, 24, 2} with respect to their accumulated regression coefficients are presented in Fig. 6b. For a better visual comparison, the histograms are scaled so that the value of the top related feature is fixed at 100.

In this following, we comprehensively discuss the significant features related to functional balance in terms of variable importance. A higher relative importance value indicates the more importance of this feature to the balance estimation. All of our findings are supported by the scientific and clinical evidences provided in existing literatures. The in-depth identification of these variables is helpful to determine the underlying causes of balance deficit. Step length is a useful clinical indicator of mobility, balance, and fall risk in elderly [45]. It has excellent discriminative power to differentiate subjects when assessing mobility dysfunction in elderly [46]. Elderly gender is another significant risk factor related to falls [47] and the frequency of falls [48]. Gait speed has been repeatedly reported to reflect functional status and has been recommended as a potentially useful clinical predictor of falls [49]. Differences in step length and gait speed suggest that elderly adopt a conservative and cautious gait pattern, possibly in an attempt to minimize the displacement of the upper body related to their balance impairments. The remaining features are linear acceleration features describe the shape and pattern of liner movements over time. Those measurements are directly related to the motor coordination deficits as a large compensatory hip and trunk motions are required to correct dis-equilibrium. The findings support that acceleration variation can be a good indicator of balance estimation. As for the walking, the root mean square of linear acceleration has been reported to measure gait smoothness, with larger values linked to increased fall risk [50, 51]. For the SiSt and StSi transitions, these linear acceleration features are directly related to the forces needed to perform the postural transitions thus explain certain impairments. This finding is consistent with previous literatures [34, 52, 53] on the study of postural transition.

### SFBBS subtask prediction

**Fig. 7:**
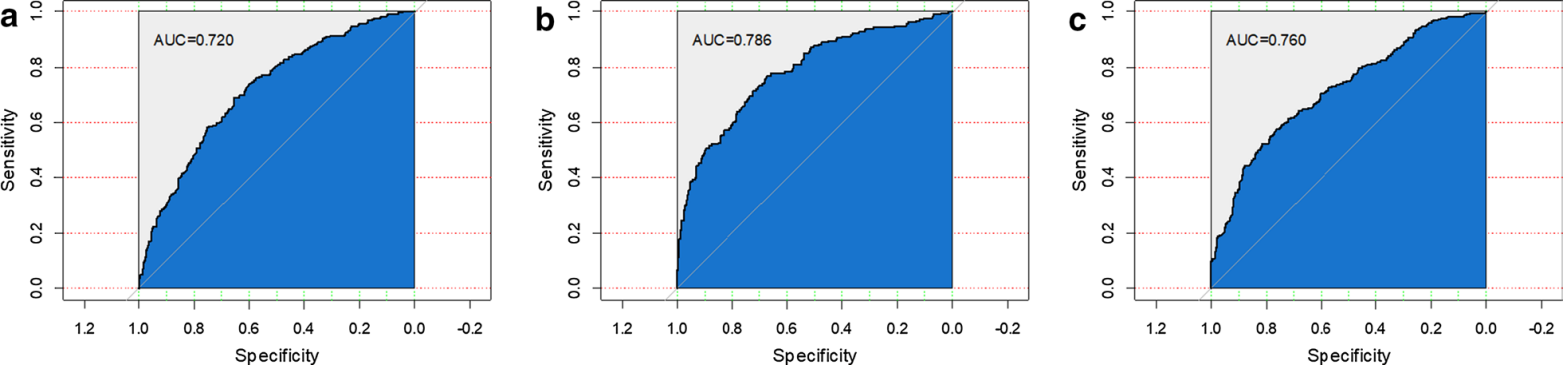
The ROC curves of classifications: **a** SFBBS-3, **b** SFBBS-6, and **c** SFBBS-7

Table 4 shows the performance of the classifications in a combination of SFBBS subtask numbers and evaluation metrics. To compare our method performance with existing ones as much as possible, here we consider a closely related problem, the fall detection. In [54], the authors benchmarked the performance of published accelerometer-based fall-detection methods when they were applied to identifying real-world falls. It was found that the sensitivity average of the thirteen studied algorithms, was (mean ± std) 83.0% ± 30.3% (maximum value = 98%), and the specificity was considerably lower (57.0% ± 27.3%, maximum value = 82.8%). This supports the effectiveness of our approach. Our study shows the average of sensitivity and specificity are 74% and 79% respectively among 3 classification tasks, which lies between the boundaries. Figure 7 further shows the ROC curves for each of the classification tasks. From a clinical perspective, it is more meaningful to use ROC curves to identify optimally sensitive and specific cut-off values which can be used to classify the elderly as being at high or low-risk groups [55]. A highly sensitive and specificity test for measuring balance is a necessity in practice since an assessment that incorrectly overlooks elderly who are actually at risk or identify elderly who are at low risk being classified at high risk would be undesirable for both clinician and elderly, considering the potential consequences of falls. The AUC values for SFBBS-3, SFBBS-6, and SFBBS-7 are 0.72, 0.79, and 0.76 respectively, which represents moderate-high discriminations.

**Table 4:** Classification results regarding SFBBS subtasks using elastic net regression

SFBBS subtask	Accuracy	Sensitivity	Precision	Specificity	F-score
SFBBS-3	0.75	0.65	0.47	0.79	0.52
SFBBS-6	0.84	0.85	0.43	0.83	0.53
SFBBS-7	0.71	0.72	0.83	0.75	0.76

### Significant features for SFBBS subtasks prediction

Herein we report and discuss the significant features in terms of their selection frequency and relative importance for each classification task.

**Fig. 8:**
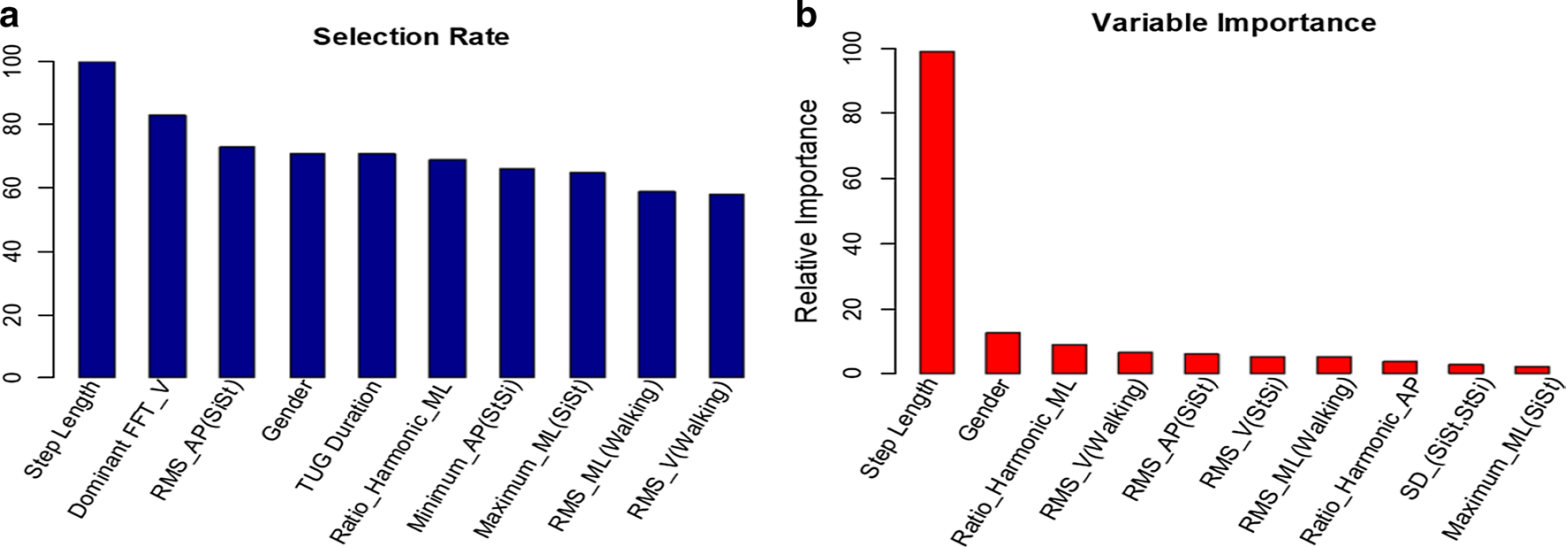
Histogram of selected features by elastic net regression using 100-RSSCV on SFBBS-3 classification: **a** top ten most selected features; **b** top ten most significant features with respect to their accumulated regression coefficients

*SFBBS-3:* Reaching forward task measures the maximal distance a subject could reach forward horizontally with outstretched arms when maintaining stable standing. The maximal distance of reached forward is considered as a reliable parameter for evaluating dynamic balance capacity [56]. Elderly showed dynamic balance control problem influenced by the weakened muscle strength in lower limbs, the tibialis anterior, soleus and gastrocnemius included. The top ten most frequently occurring features in descending order of frequency are {27, 34, 13, 67, 1, 41, 48, 3, 25, 24}, whereas the top ten most relative features are {27, 67, 41, 24, 13,52, 52, 25, 42, 65, 3}, shown in Fig. 8.

**Fig. 9:**
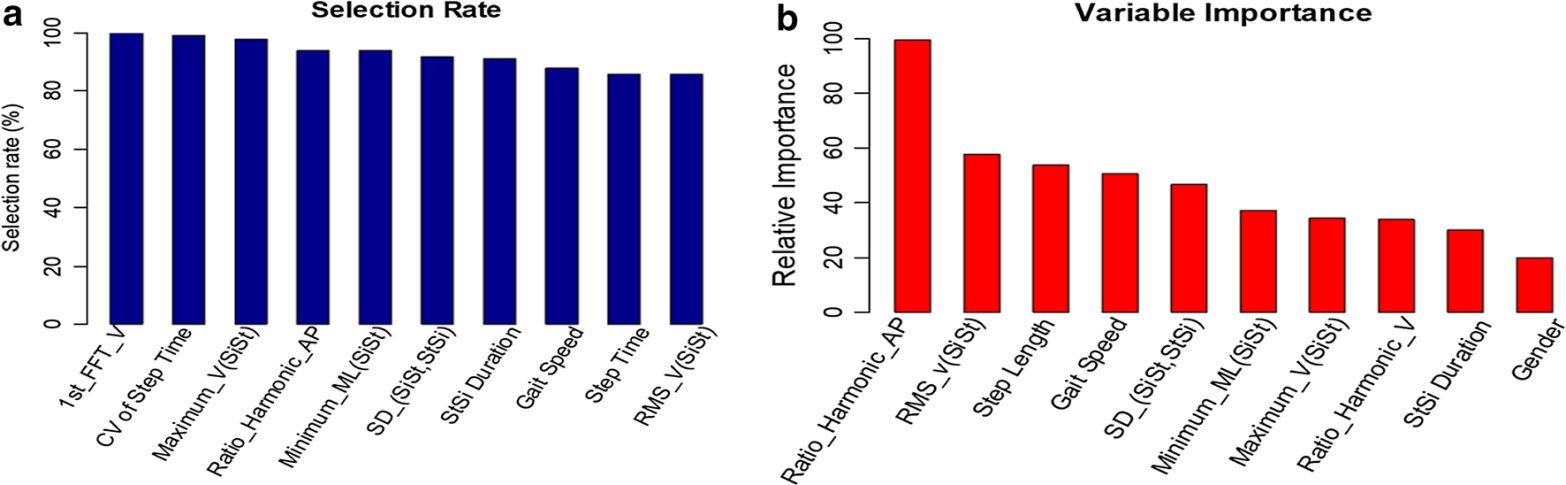
Histogram of selected features by elastic net regression using 100-RSSCV on SFBBS-6 classification: **a** top ten most selected features; **b** top ten most significant features with respect to their accumulated regression coefficients

*SFBBS-6:* Standing unsupported measures a subject’s muscle strength, joint range-of-motion, and body coordination to maintain equilibrium. Unlike younger people who are usually able to counteract imbalance preferably with an ankle strategy, elderly tend to do so with a hip strategy [57]. The top ten most frequently occurring features in descending order of frequency are {37, 32, 2, 42, 6, 65, 64, 29, 30, 11}, whereas the top ten most relative features are {42, 11, 27, 29, 65, 6, 2, 40, 64, 67}, shown in Fig. 9.

**Fig. 10:**
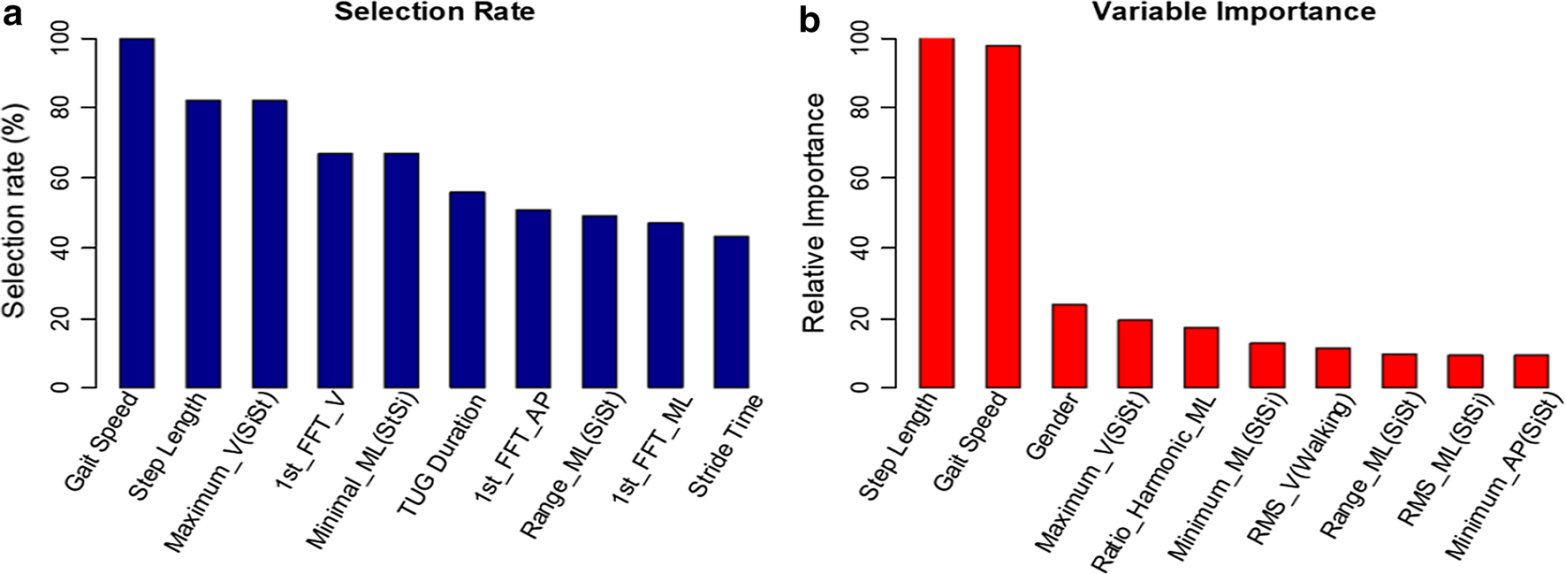
Histogram of selected features by elastic net regression using 100-RSSCV on SFBBS-7 classification: **a** top ten most selected features; **b** top ten most significant features with respect to their accumulated regression coefficients

*SFBBS-7:* One-leg standing test measures a subject’s ability to stand on one lower limb without support. It accesses postural steadiness in a static stance. The previous research has revealed one-leg standing time depends on the ability of somatosensory and vision functions [58]. Lack of accurate feedback and diminished ankle strategy can result in postural instability, especially in ML direction [59]. The top ten most frequently occurring features in descending order of frequency are {29, 27, 2, 37, 47, 1, 39, 9, 38, 31}, whereas the top ten most relative features are {27, 29, 67, 2, 41, 47, 24, 9, 53, 7}, shown in Fig. 10.

Similar to the contributed variables to the SFBBS score prediction, step length, gender, gait speed, and linear acceleration variables play a significant role at different extent in the classifications of balance risk level of three selected clinical assessment task. We also find that REOH is another significant variable. REOH reflects the proportion of the acceleration signal that is in phase with an elderly’s stride frequency, where a small REOH indicates step-to-step asymmetry within strides and possibly gait instability [29].

## Conclusion

Poor balance is one of the major intrinsic factors contributing to falls, and the continuous monitoring of balance and gait is a plausible approach, through early warnings and appropriate interventions, to reduce and prevent falls. However, the continuous monitoring of gait and balance requires extensive healthcare and clinical resources. In this paper, we investigated the effectiveness of 3MTUG test via accelerometers and present a clinically relevant approach to assessing functional balance and mobility of community-dwelling elderly in terms of SFBBS. The clinical perspective of our study advances the application of wearable-sensors in the field of fall risk assessment. We summarize the main contributions of this paper as follows. This work is the first to extract variables which have previously been associated with fall-risk in elderly with an emphasis on their clinical relevance by separate consideration of postural transitions and walking phases during the TUG test. Our results demonstrate that use of the proposed clinically relevant variables, together with utilization of proper modeling and cross-validation strategies, is able to achieve a moderate-high predictive accuracy of SFBBS scores. The investigation of three clinical balance assessment tasks for identifying the balance risk level of elderly further demonstrates the efficiency and robustness of the proposed method. Our in-depth identifications of a range of significant variables enable the diagnosis and early treatment of balance impairment. The research setting, as well as the proposed method, presents a feasible option for objective, unsupervised, unobtrusive balance assessment at the point-of-care or home environment. The proposed automatic estimation for quantifying balance during prescribed task provides clinicians and elderly with accurate, stable, and sensitive biomarkers for long-term monitoring of functional balance. The diagnostics of balance deficit will facilitate timely intervention before a fall occurs and, hence improve quality of life and avert the need for a higher-level intervention in the future.

While this study clearly demonstrates the utility of a 3MTUG walking test for accessing an elderly’s functional balance and mobility, future work will focus on developing a more effective and robust surrogate assessment of functional balance. Many anthropometric factors have been previously studied to have effect on balance, the inclusion of these variables need to be investigated for the enriched automatic assessment. Besides, future work could examine the effectiveness of the integrated use of other sensing modalities (such as gyroscope) in adopting our surrogate assessment of functional balance. This additional information provides a more comprehensive characterization of movement adaptation, which may, in turn, further improve the robustness of the surrogate assessment.

## Data Availability

The datasets generated and analyzed during the current study are not publicly available due to Institutional Review Board related matters, but are available from the corresponding authors (Yang Zhao zhaoyang_elsa@163.com or Kwok-Leung Tsui kltsui@vt.edu) on reasonable request.

## Abbreviations

3MTUG: 3-M timed up and go
AP: Anterior–posterior
AUC: Area under the curve
BBS: Berg balance scale
COM: Center-of-mass
CV: Coefficient of variation
DR: Directed routine
FFT: Fast Fourier transformation
GLM: Generalized linear model
ICC: Intra-class correlation coefficient
MAE: Mean absolute error
ML: Medial–lateral
OLS: Ordinary least squares
PPA: Physiological profile assessment
REOH: Ratio of even to odd harmonics
RMSE: Root mean square error
ROC: Receiver operating characteristic
RSSCV: Random shuffle split cross validation
SFBBS: Short form berg balance scale
SiSt: Sit-to-stand
StSi: Stand-to-sit
TUG: Timed up and go
V: Vertical
